# Decreased Siglec-9 Expression on Natural Killer Cell Subset Associated With Persistent HBV Replication

**DOI:** 10.3389/fimmu.2018.01124

**Published:** 2018-05-30

**Authors:** Di Zhao, Xuemei Jiang, Yong Xu, Huimin Yang, Dongni Gao, Xueen Li, Lifen Gao, Chunhong Ma, Xiaohong Liang

**Affiliations:** ^1^Key Laboratory for Experimental Teratology of Ministry of Education, Key Laboratory of Infection and Immunity of Shandong Province and Department of Immunology, Shandong University School of Medicine, Jinan, China; ^2^Department of Clinical Laboratory, Qilu Hospital of Shandong University, Jinan, China; ^3^Department of Hepatic Diseases, Jinan Infectious Disease Hospital, Jinan, China; ^4^Department of Nephrology, Qilu Hospital, Shandong University, Jinan, China; ^5^Department of Neurosurgery, Qilu Hospital, Shandong University, Jinan, China

**Keywords:** chronic hepatitis B, Siglec-9, NK, cytokine synthesis, cytotoxicity

## Abstract

Siglec-9 is an MHC-independent inhibitory receptor selectively expressed on CD56^dim^ NK cells. Its role in infection diseases has not been investigated yet. Here, we studied the potential regulatory roles of NK Siglec-9 in the pathogenesis of chronic hepatitis B (CHB) infection. Flow cytometry evaluated the expression of Siglec-9 and other receptors on peripheral NK cells. Immunofluorescence staining was used to detect Siglec-9 ligands on liver biopsy tissues and cultured hepatocyte cell lines. Siglec-9 blocking assay was carried out and cytokine synthesis and CD107a degranulation was detected by flow cytometry. Compared to healthy donors, CHB patients had decreased Siglec-9^+^ NK cells, which reversely correlated with serum hepatitis B e antigen and HBV DNA titer. Siglec-9 expression on NK cells from patients achieving sustained virological response recovered to the level of normal donors. Neutralization of Siglec-9 restored cytokine synthesis and degranulation of NK cells from CHB patients. Immunofluorescence staining showed increased expression of Siglec-9 ligands in liver biopsy tissues from CHB patients and in hepatocyte cell lines infected with HBV or stimulated with inflammatory cytokines (IL-6 or TGF-β). These findings identify Siglec-9 as a negative regulator for NK cells contributing to HBV persistence and the intervention of Siglec-9 signaling might be of potentially translational significance.

## Introduction

Chronic hepatitis B (CHB) infection is still a major global health problem as a critical cause of liver cirrhosis and hepatocellular carcinoma (1). Worldwide more than 250 million people are chronically infected with HBV and each year a growing number of patients die of chronic HBV infection related diseases (2). Over the past several decades, a number of algorithms have been developed in an attempt to explore the mechanisms of persistent HBV replication. Thereinto, dysregulated immune response was deemed to play a crucial role in the chronicity of infection (3). NK cells, as the first line of host defense, have the capacity to recognize and lyze the infected hepatocytes or secrete cytokines to clear infection. However, in CHB patients, the percentage of liver and peripheral NK cells is reduced and the potential of cytokine secretion such as IFN-γ and TNF-α is defective (4). Mechanistic studies showed that the imbalanced expression of activating (NKp30, NKp46, NKG2D, etc.) and inhibitory receptors (NKG2A, Tim-3, etc.) closely correlates with the dysfunction of NK cells in CHB patients (5, 6). Accordingly, blocking inhibitory receptors (2, 6, 7) recovers the cytokine production and cytotoxicity of NK cells, which seems to be a promising strategy for controlling chronic infection. Thus, identification of novel NK receptors involved in CHB pathogenesis might provide potential therapeutic target.

Siglecs (sialic-acid-binding immunoglobulin-like lectins) comprise lectin family of surface receptors that are predominantly expressed on hematopoietic cells with cell type and differentiation specificity (8–11). By binding with sialic acids at the terminal position of glycoproteins and -lipids (also called sialogylcans), Siglecs regulate the intensity and duration of innate or adaptive immune response (12, 13). More recently, the involvement of Siglecs in the immune evasion of tumor cells and virus-infected cells attracts most attentions (14). Siglec-9 belongs to the CD33-related Siglec family, one major group of the Siglecs, and contains a total of three Ig domains, a V-set domain and two C2-set domains, and a cytoplasmic region containing ITIM-like and SLAM-like motifs (15). Siglec-9 is expressed on granulocytes, monocytes, B cells, CD56^+^ NK cells, and weakly on minor subsets of CD8^+^ and CD4^+^ T cells (16, 17). Siglec-9 prefers to bind with sialic acid α2,3 linked to galactose, recruits the tyrosine phosphatases SHP-1 and SHP-2 and executes its regulatory functions on multiple immune cells (8, 15). It was reported that cross-linking of Siglec-9 in neutrophils not only helps maintain its quiescence in the bloodstream (18) but also suppresses its recruitment, oxidative burst, and induces cell death in tumor or inflammatory milieu (19–21). Besides, the mucin MUC1 containing multiple short, sialylated O-linked glycans (MUC1-ST) on tumor cells facilitate the tumor-associated macrophages to acquire the protumoral phenotype by binding with Siglec-9 receptor (22). For NK cells, Siglec-9 also executes important functions. It was reported that Siglec-9 is selectively expressed in CD56^dim^ NK cells (23) and the presence of Siglec-9 defines a subset of NK cells with a mature phenotype and enhanced chemotactic potential (24). However, in cancer patients, Siglec-9^+^ NK cell population was reduced (24). All these findings suggest that Siglec-9 is emerging as an important immune regulator under either physiological or pathological conditions. However, the role of Siglec-9 in viral infection remains unknown yet.

In the present study, we for the first time demonstrates that decreased Siglec-9 expression on NK cells in CHB patients correlates with HBV viremia. Blocking of Siglec-9 reverses NK cells suppression, which might be a novel potential way to control persistent HBV replication.

## Materials and Methods

### Patients

Cubital venous blood was collected from 79 untreated patients with chronic HBV infection and 59 healthy donors (HDs). The patients’ demographic, clinical, and laboratory characteristics are listed in Table 1. The diagnosis of CHB patients was made according to the criteria established in the National Viral Hepatitis Conference of China (2000). Those subjects who were infected with hepatitis C virus (HCV), hepatitis D virus, hepatitis G virus, and HIV or attacked with autoimmune liver diseases were all excluded. Follow-up samples were available from 20 patients with chronic HBV infection who achieved sustained virological response (SVR) after antiviral therapy. Sera level of hepatitis B e antigen (HBeAg) was analyzed with commercial Enzyme Immunoassay kits (Kewei Diagnostic, Beijing, China). The level of serum HBV DNA was quantified using HBV diagnostic kit (PG Biotech, LTD., Shenzhen, China). Informed consent was obtained from all patients before the study was initiated with approval of the Shandong University Medical Ethics Committee in accordance with the Declaration of Helsinki.

**Table 1:** Clinical characteristics of human samples.

Sample characteristics	Group1 (CHB patients without treatment)	Group2 (CHB patients achieving SVR)	Group3 (healthy donors)
Number of patients	79	20	59
Age (years; median, range)	(41, 50)	(51, 38)	(33, 54)
Gender (male/female)	(52/27)	(14/6)	(37/22)
HBeAg (negative/positive)	(35/44)	(20/0)	(59/0)
HBV DNA (IU/ml; >10^3^/<10^3^)	(50/29)	(0/20)	(0/59)
HBsAg (negative/positive)	(0/79)	(0/20)	(59/0)
HBsAb (negative/positive)	(79/0)	(20/0)	(0/59)
ALT (U/l; ≤42/>42)	(37/42)	(20/0)	(59/0)

*HBsAg, hepatitis B surface antigen; HBeAg, hepatitis B e antigen; HBsAb, hepatitis B surface antibody; CHB, chronic hepatitis B; SVR, sustained virological response*.

### Flow Cytometric Analysis

Peripheral whole blood cells were incubated with PerCp/cy5.5 anti-human CD3, PE/Cy7 anti-human CD56, PE anti-human Siglec-9, APC/Cy7 anti-human NKG2D (CD314), FITC anti-human CD335 (NKp46), APC anti-human CD337 (NKp30) (BioLegend, San Diego, CA, USA), and APC anti-human NKG2A (Miltenyi Biotech GmbH, Bergisch Gladbach, Germany) for 30 min and submitted to RBC lysis using FACS lysis solution (BD Biosciences, San Jose, CA, USA). The stained cells were analyzed using a BD FACS Canto II Flow Cytometer and the data were analyzed using BD FACS Diva software (BD Biosciences, San Jose, CA, USA).

### PBMCs Isolation, Activation, and Siglec-9 Neutralization

Whole blood samples were collected from CHB patients after obtaining informed consent. PBMCs were obtained by density gradient centrifugation on Ficoll-Hypaque (TBD science, Tianjin, China), according to the manufacturer’s protocol. For Siglec-9 blockade assay, PBMCs were preincubated with human Siglec-9 antibody with a final concentration of 10 µg/ml or IgG controls (R&D, Minneapolis, MN, USA) for 40 min and then stimulated with PMA (50 ng/ml) (SIGMA, St. Louis, MO, USA), ionomycin (1 µg/ml) (BioLegend, San Diego, CA, USA), and Brefeldin A (10 µg/ml) (BioLegend, San Diego, CA, USA) for 4 h. After incubation with PerCp/cy5.5 anti-human CD3, PE/Cy7 anti-human CD56, cells were treated with the permeable agent Cytofix/Cytoperm (eBioscience, San Diego, CA, USA) according to the manufacturer’s instructions and were intracellularly stained with FITC anti-human IFN-γ (Miltenyi Biotech GmbH, Bergisch Gladbach, Germany) or APC anti-human TNF-α (eBioscience, San Diego, CA, USA) for 30 min. The expression of IFN-γ and TNF-α were analyzed by flow cytometry. To assess the degranulation ability, PMBCs were stimulated with PMA and ionmycin plus BFA in the presence preincubated with PE anti-human CD107a (BioLegend, San Diego, CA, USA) for 4 h. Then the cell surface CD107a expression was detected by flow cytometry.

### Detection of Siglec-9 Ligand on Liver Biopsy Tissues and Hepatocyte Cells

Siglec-9 ligand expression was detected by immunofluorescence staining in paraffin-embedded liver biopsy tissue (5 µm) from CHB patients or normal liver tissues from surgery, or in hepatocyte cell lines infected with HBV particles or stimulated with cytokines enriched in HBV-infected liver microenvironment (IL-6 or TGF-β) (25, 26). Briefly, recombinant human Siglec-9 Fc (R&D Systems, Minneapolis, MN, USA) were mixed with PE-conjugated anti-human Ig (Jackson ImmunoResearch Laboratories, West Grove, PA, USA) for 1 h at 4°C before use. Images were taken using a confocal laser microscope (Carl Zeiss, LSM780, Oberkochen, Germany).

### Statistical Analysis

All data were analyzed using the GraphPad Prism 5. The Mann–Whitney nonparametric *U* tests and Student’s *t* test were used for comparison between groups. Spearman correlation analysis was performed between the Siglec-9 expression and clinical data. A *p* < 0.05 is considered as statistically significant.

## Results

### CHB Patients Have Decreased Siglec-9 Expression on NK Cells

**Figure 1:**
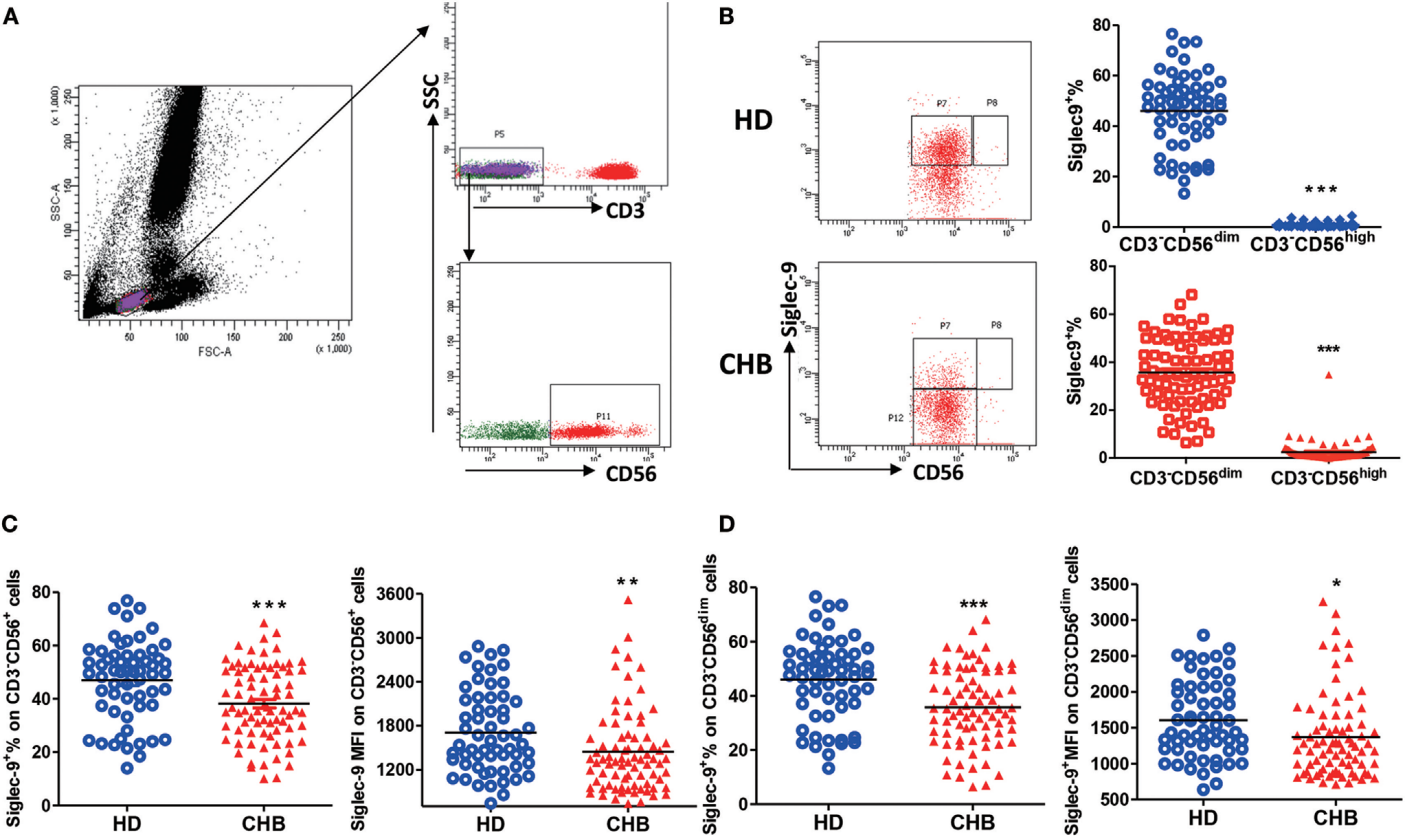
Decreased Siglec-9 expression on NK cells from chronic hepatitis B (CHB) patients. **(A)** The gating strategy for identification of lymphocytes, CD3^−^ cells, and CD3^−^CD56^+^ NK cells. **(B)** The percentage of Siglec-9 expression on CD3^−^CD56^dim^ NK cells and CD3^−^CD56^high^ NK cells from both healthy donors (HDs) and CHB patients. Representative dot plots were shown in left panels. **(C)** The percentage and mean fluorescence intensity (MFI) of Siglec-9 expression on CD3^−^CD56^+^ NK cells. **(D)** The percentage and MFI of Siglec-9 expression on CD3^−^CD56^dim^ NK cells.

Siglec-9 expression on CD3^−^CD56^+^ NK cells was examined in 79 untreated patients with chronic HBV infection and 59 HDs (Figure 1A; Figure S1 in Supplementary Material). Consistent with the literature (24), there was moderate Siglec-9 expression on CD3^−^CD56^+^ NK cells (Figure 1B). It is well-known that human CD3^−^CD56^+^ NK cells can be categorized into two functionally distinct subsets based on relative CD56 expression: CD56^bright^ and CD56^dim^ (4). Thus, we further analyzed the difference in Siglec-9 expression in these two subsets. We found that Sigec-9 was especially expressed on CD3^−^CD56^dim^ NK cells in both HDs and CHB patients (Figure 1B). However, compared with HDs, CHB patients had significantly decreased Siglec-9 percentage and mean fluorescence intensity (MFI) on circulating CD3^−^CD56^+^ NK cells (Figure 1C). This disparity between HD and CHB patients was also validated in CD3^−^CD56^dim^ NK cells (Figure 1D). These findings indicate that Siglec-9 on NK cells might involve in the pathogenesis of CHB.

### Siglec-9 Expression on NK Cells Is Reversely Correlated With Virus Replication Status in CHB Patients

**Figure 2:**
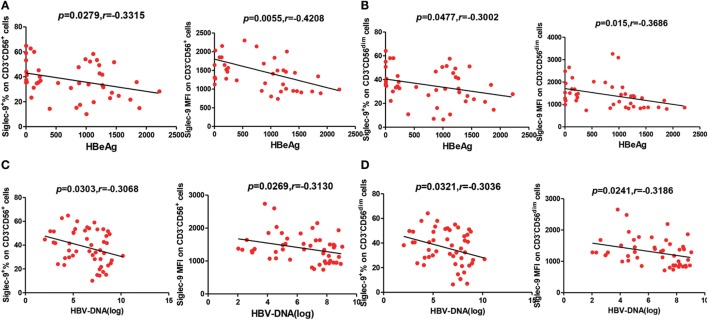
Negative correlation between Siglec-9 expression on NK cells and HBV viremia in chronic hepatitis B (CHB) patients. **(A,C)** Association of Siglec-9 expression on CD3^−^CD56^+^ NK cells with serum hepatitis B e antigen (HBeAg) levels and HBV DNA in CHB patients. *p* Values are shown. **(B,D)** Association of Siglec-9 expression on CD3^−^CD56^dim^ NK cells with serum HBeAg levels and HBV DNA in CHB patients. *p* Values are shown.

Considering the important roles of NK cells in antiviral immunity, we further analyzed the correlation of Siglec-9 expression on NK cells with the viremia level of CHB patients. As shown in Figure 2A, the concentration of serum HBeAg, an indicator of HBV DNA replication, in CHB patients negatively correlated with the percentage and MFI of Siglec-9 on circulating CD3^−^CD56^+^ NK cells. Similar correlation was found between HBeAg concentration and Siglec-9 expression on CD3^−^CD56^dim^ NK cells (Figure 2B). In addition, we further determined the negative correlation of serum HBV DNA level with Siglec-9 expression on CD3^−^CD56^+^ NK cells (Figure 2C) and CD3^−^CD56^dim^ NK cells (Figure 2D).

**Figure 3:**
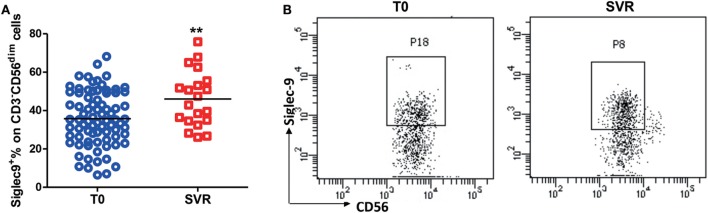
Restored Siglec-9 expression on NK cells in chronic hepatitis B (CHB) patients achieving sustained virological response (SVR). **(A)** The percentage of Siglec-9 expression on CD3^−^CD56^dim^ NK cells in CHB patients without treatment (T0) and in patients achieving SVR. **(B)** Representative dot plots were shown.

To further validate the relationship between Siglec-9^+^ NK cells and the persistence of HBV replication, we analyzed Siglec-9 expression in CHB patients without clinical treatment and patients achieving SVR (no detectable viremia) after antiviral therapy. As shown in Figures 3A,B, Siglec-9 expression on NK cells was significantly upregulated after SVR. Together, these results suggest that CD56^dim^Siglec-9^+^ NK cells might play a role in controlling HBV replication.

### Siglec-9 Positive NK Cells in CHB Patients Exhibit More Activating Phenotype

**Figure 4:**
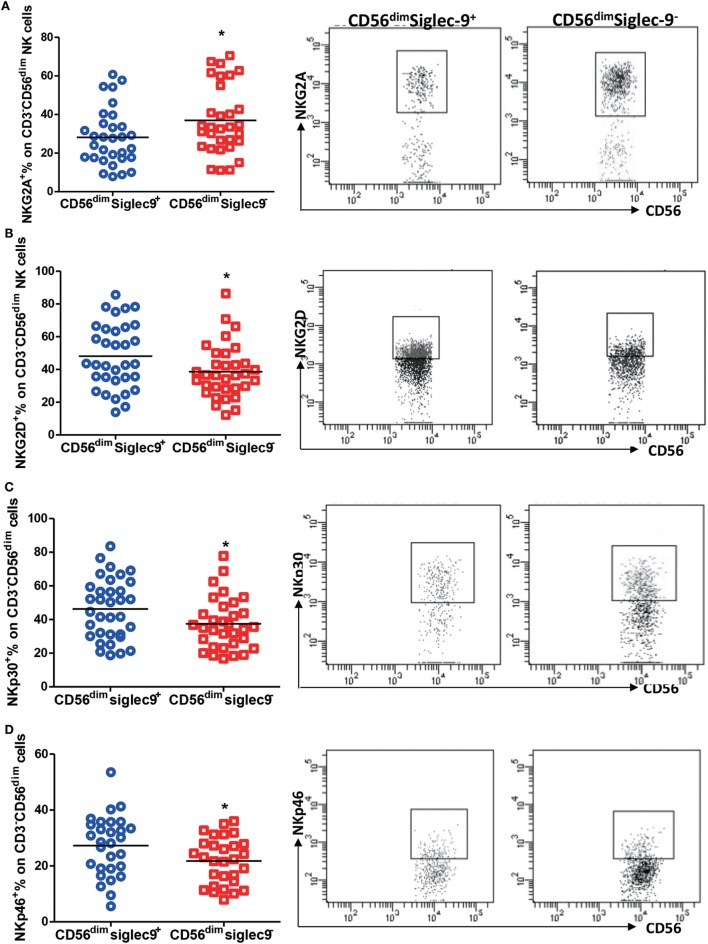
Siglec-9 positive NK cells shows more activating phenotype in chronic hepatitis B patients. **(A–D)** The expression of NKG2A **(A)**, NKG2D **(B)**, NKp30 **(C)**, and NKp46 **(D)** in Siglec-9^+^CD3^−^CD56^dim^ NK cells and Siglec-9^−^CD3^−^CD56^dim^ NK cells. Representative dot plots were shown in right panel.

As reported that dysregulated expression of NK receptors on cells largely contributes to its dysfunction in tumors and chronic infection (5), we thus evaluated the expression of NKG2D, NKG2A, NKp30, and NKp46 on Siglec-9 positive and negative NK cells from CHB patients. Flow cytometry analysis showed that Siglec-9 positive CD56^dim^ NK cells expressed lower level of NKG2A (one of important inhibitory receptors on NK cells) (Figure 4A), but higher level of several activating receptors (NKG2D, NKp30, and NKp46) (Figures 4B–D), than Siglec-9 negative cells. Similarly, in HDs, Siglec-9 positive cells showed higher expression of NKp30 (Figure S2 in Supplementary Material). These results clue that CD56^dim^Siglec-9^+^ NK cells displayed more activating phenotype and the decrease of this NK cell subtype might be responsible for defective antiviral immunity in CHB patients.

### Siglec-9 Blockade Restores NK Cell Function in CHB Patients

**Figure 5:**
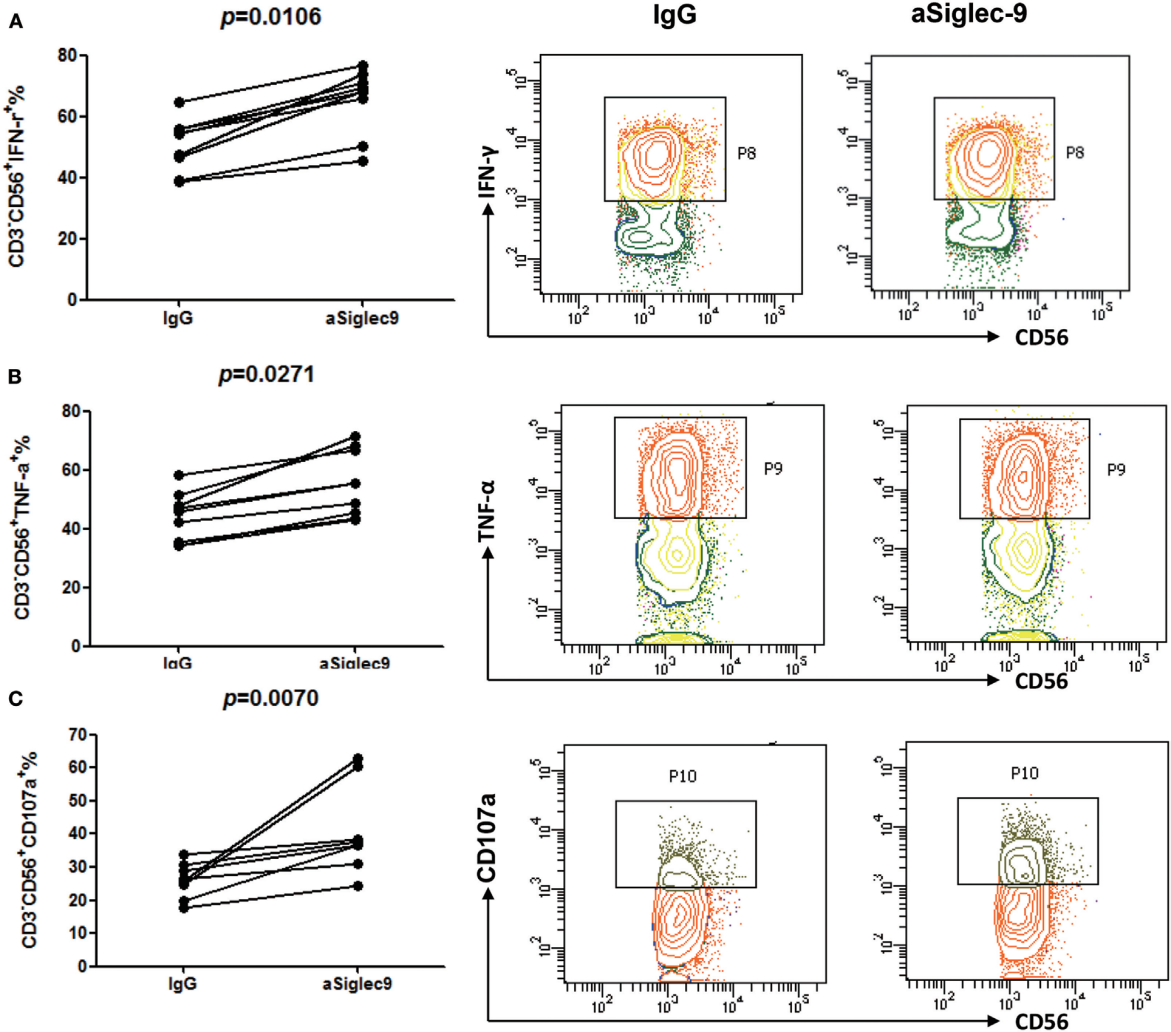
Siglec-9 blockade restores cytokine secretion and degranulation of NK cells in chronic hepatitis B (CHB) patients. PBMCs from CHB patients pretreated with Siglec-9 blocking antibody or isotype control IgG were stimulated with PMA plus ionomycin. Then, the expression of IFN-γ **(A)**, TNF-α **(B)**, and CD107a **(C)** were analyzed by flow cytometry. The right panels showed the representative flow cytometry data from one subject.

In order to estimate the role of Siglec-9 on NK cells in the pathogenesis of CHB, PBMCs from CHB patients were pretreated with Siglec-9 neutralizing antibody and then stimulated with PMA plus ionomycin. Flow cytometry analysis demonstrated that compared to IgG control cells, pretreatment of PBMCs with Siglec-9 blocking antibody significantly increased IFN-γ and TNF-α expression (Figures 5A,B) and CD107a degranulation (Figure 5C) of NK cells. We also found Siglec-9 blockade improved cytokine production in NK cell line NK-92 (Figure S3 in Supplementary Material). However, we did not observe any obvious effect of Siglec-9 blockade on cytokine production and degranulation ability of NK cells from HDs (Figure S4 in Supplementary Material). These results suggest that Siglec-9 involves in the dysregulation of NK cells in hepatitis B infection.

### Elevated Siglec-9L Level in HBV Infection and Inflammatory Milieu

**Figure 6:**
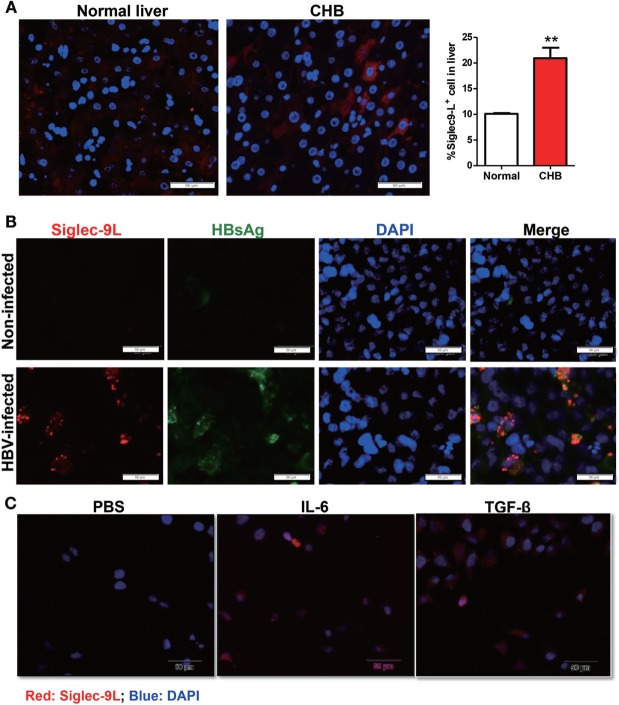
Upregulated expression of Siglec-9 ligand in HBV infection and inflammatory milieu. **(A)** Detection of Siglec-9 ligand in liver biopsies from chronic hepatitis B (CHB) patients or normal liver tissues by immunofluorescence using Siglec-9-Fc chimera protein. **(B,C)** Expression of Siglec-9 ligand and hepatitis B surface antigen (HBsAg) in hepatoma cell line HLCZ01 with or without HBV infection **(B)**, and Huh7 cells treated with 100 ng/ml IL-6 or TNF-α for 24 h **(C)**, was evaluated by immunofluorescence staining.

Considering that Siglec-9 signaling transduction requires its binding to glycoproteins or -lipids ligands, we thus analyzed the expression of Siglec-9 ligand in liver biopsies from CHB patients or normal liver tissues by immunofluorescence using Siglec-9-Fc chimera protein. As shown in Figure 6A, increased expression of Siglec-9 ligand were seen along the cell periphery as discontinuous punctuate spots in liver biopsy tissues of CHB patients compared with that in normal liver tissues. The same expression pattern of Siglec-9 ligand was also found in HBV-infected HLCZ01 cells, which was significantly higher than that in non-infected control cells (Figure 6B). Importantly, we observed the colocalization of hepatitis B surface antigen and Siglec-9L in HBV-infected HLCZ01 cells. In addition, the binding of Siglec-9-Fc chimera protein with Huh7 cells was also increased by cytokines (IL-6, TGF-β, etc.) (Figure 6C), both of which are upregulated in HBV-infected cells (Figure S5 in Supplementary Material). These findings indicate that HBV itself and cytokines in liver microenvironment involve in elevating Siglec-9 ligand on hepatocytes, which might explain the dysregulation of NK cells in CHB patients.

## Discussion

Siglecs are emerging inhibitory immunoregulatory molecules that control host immune responses *via* interactions with glycans ligands. Accumulating literatures disclosed that Siglecs may play important roles in diseases, e.g., infectious diseases, inflammation, autoimmunity, and cancer, making them attractive targets for clinical intervention (27).

Siglec-9, broadly expressed on leukocytes, regulates the intensity of innate and adaptive immunity. Especially, Siglec-9 defines a subset of CD56^dim^ NK cells with a mature phenotype (24). However, in cancer patients, Siglec-9^+^ NK cell population in peripheral blood was reduced (24). Till now, the significance of Siglec-9 in virus infection remains unknown. Here, we found that similar to that in HDs, Siglec-9 was dominantly expressed in CD56^dim^ NK cells in CHB patients, while CD56^bright^ NK cells only possessed barely detectable expression. Nevertheless, Siglec-9^+^ NK cells were significantly reduced in CHB patients. Previous studies have shown reduced NK number and defective NK functions involve in the persistence of HBV replication and the pathogenesis of CHB (4). We also found decreased NK number in liver biopsies of CHB patients (Figure S6 in Supplementary Material). Importantly, we found that the percentage of Siglec-9^+^ NK cells and the MFI of Siglec-9 on NK cells negatively correlated with serum HBeAg level and HBV DNA level. More interestingly, this subset of NK cells seems to recover in patients achieving SVR (no detectable viremia) after antiviral therapy. Our findings clearly demonstrate that the status of HBV infection might have a direct association with the ratio of Siglec-9^+^ NK cells in peripheral blood of CHB patients. However, the regulatory relationship between HBV infection and Siglec-9 on NK cells needs to further investigation.

It is well accepted that the function of NK cells are finely controlled by the panel of surface receptors (28). Of note, our results showed that in CHB patients, Siglec-9^+^ NK cells showed a more activated phenotype, with higher expression of activating receptors (NKG2D, NKp30, and NKp46) and lower expression of inhibitory receptor (NKG2A). This expression pattern is similar to some of other inhibitory receptors (e.g., PD-1), which might play a role in mediating exhaustion of activated NK cells (29). Siglecs, as a class of ITIM-containing, MHC-independent inhibitory receptors, transmit negative signals into NK cells even when the target cells lose the expression of MHC class I molecule (missing-self) or when the classical inhibitory NK receptors are inefficiently engaged. Thus, Siglecs are exploited by tumor cells or virus-infected host cells to evade the host immune surveillance and manipulation of this pathway might be a strategy to circumventing immune escape. Here, we found that blockade of Siglec-9 led to increased intracellular IFN-γ and TNF-α expression and CD107a degranulation of NK cells from patients with CHB, preliminarily attesting its prospect in immunotherapy of CHB infection.

The ligation to specific glycoprotein or -lipids is prerequisite of Siglecs signaling in immune cells. Indeed, overexpressed broad spectrum (24) or specific sialic acid-containing ligands (e.g., MUC16, GD3, GD2, GT1b, etc.) are reported to be responsible for the impaired immune status in tumor microenvironment and serves as prognostic markers and therapy targets (23, 30, 31). Some specific structures on pathogens also bind with Siglecs [e.g., the interaction between HCV E2 envelope protein and Siglec-7 (32)] and in turn modulates anti-infection immunity. Although studies showed that differential expression of sialic acid in serum have a prognostic value for CHB patients (33), the expression pattern of sialic acid-containing ligands in HBV-infected hepatocytes remains unknown. Here, we detected increased expression of Siglec-9 ligands in liver biopsy tissues of CHB patients. Further analysis showed that both HBV infection and cytokines (IL-6 and TGF-β) upregulated Siglec-9L on cultured HCC cell lines. These results clue that in HBV-infected liver microenvironment, elevated level of Siglec-9 ligands might participate in modulating local immune status and contribute to the persistence of infection. However, identification of specific sialoglycans binding to Siglec-9 needs further pull-down assay.

In conclusion, our findings discussed here may have a broader universal significance since an enrichment of dysfunctional Siglec-9^neg^ NK cells, as described in tumor patients, also appear in patients with CHB, which might represent a novel mechanism for virus-induced NK anergy. Moreover, evidence that neutralization of Siglec-9 by blocking antibody restores cytokine synthesis and cytotoxic capability of NK cells is of potentially important translational significance.

## Ethics Statement

This study was carried out in accordance with the recommendations of the Shandong University Medical Ethics Committee with written informed consent from all subjects. All subjects gave written informed consent in accordance with the Declaration of Helsinki. The protocol was approved by the Shandong University Medical Ethics Committee.

## Author Contributions

DZ designed and took part in the implementation of all experiments, analysis, and interpretation of the analysis within this paper. XJ helped collect samples and helped with the clinical analysis. YX helped with the flow cytometry analysis. HY helped with the detection of Siglec-9 ligand on liver biopsy tissues and hepatocyte cells. DG helped the collection of samples. XuL helped with the clinical analysis and the preparation of the manuscript. LG helped with Siglec-9 neutralization design and statistical analysis for all the experiments. XiL and CM are the principle investigator and senior author for this project.

## Conflict of Interest Statement

The authors declare that the research was conducted in the absence of any commercial or financial relationships that could be construed as a potential conflict of interest.
