# Supplementary material for: Decreased Siglec-9 Expression on Natural Killer Cell Subset Associated With Persistent HBV Replication
Source: Front Immunol. 2018 May 30;9:1124. doi: 10.3389/fimmu.2018.01124 (PMC5988867; doi:10.3389/fimmu.2018.01124)
Supplement: Supplementary file 1 [file Data_Sheet_1.DOCX]

**Supplementary figure legends:**

**Supplementary figure 1. FMO control for Siglec-9, CD56 and CD3.**

**Supplementary figure 2. NK receptor expression on Siglec-9 positive and negative NK cells from HD.** (A-D) The expression of NKG2A (A), NKG2D (B),NKp30 (C), and NKp46 (D) in siglec9+CD3-CD56dim NK cells and siglec9-CD3-CD56dim NK cells. Representative dot plots were shown in right panel.

**Supplementary figure 3. Siglec-9 blockade restores cytokine secretion NK 92 cells.** NK92 cells pretreated with Siglec-9 blocking antibody or isotype control IgG were stimulated with PMA plus ionomycin. Then, the expression of IFN-γ (A) and TNF-α (B) of were analyzed by flow cytometry. The right panels showed the representative flow cytometry data from one subject.

**Supplementary figure 4. Siglec-9 blockade had no significant effect on cytokine secretion and degranulation of NK cells in HD.** PBMCs from HD pretreated with Siglec-9 blocking antibody or isotype control IgG were stimulated with PMA plus ionomycin. Then, the expression of IFN-γ (A), TNF-α (B) and CD107a (C) of were analyzed by flow cytometry. The right panels showed the representative flow cytometry data from one subject.

**Supplementary figure 5. Expression of IL-6 and TGF-β in hepatoma cell line HLCZ01 with or without HBV infection.**

**Supplementary figure 6.** Immunofluorescence staining for CD56 in normal liver tissues and liver biopsies from CHB patients
