# Supplementary figures and images for: Decreased Siglec-9 Expression on Natural Killer Cell Subset Associated With Persistent HBV Replication
Source: Front Immunol. 2018 May 30;9:1124. doi: 10.3389/fimmu.2018.01124 (PMC5988867; doi:10.3389/fimmu.2018.01124)

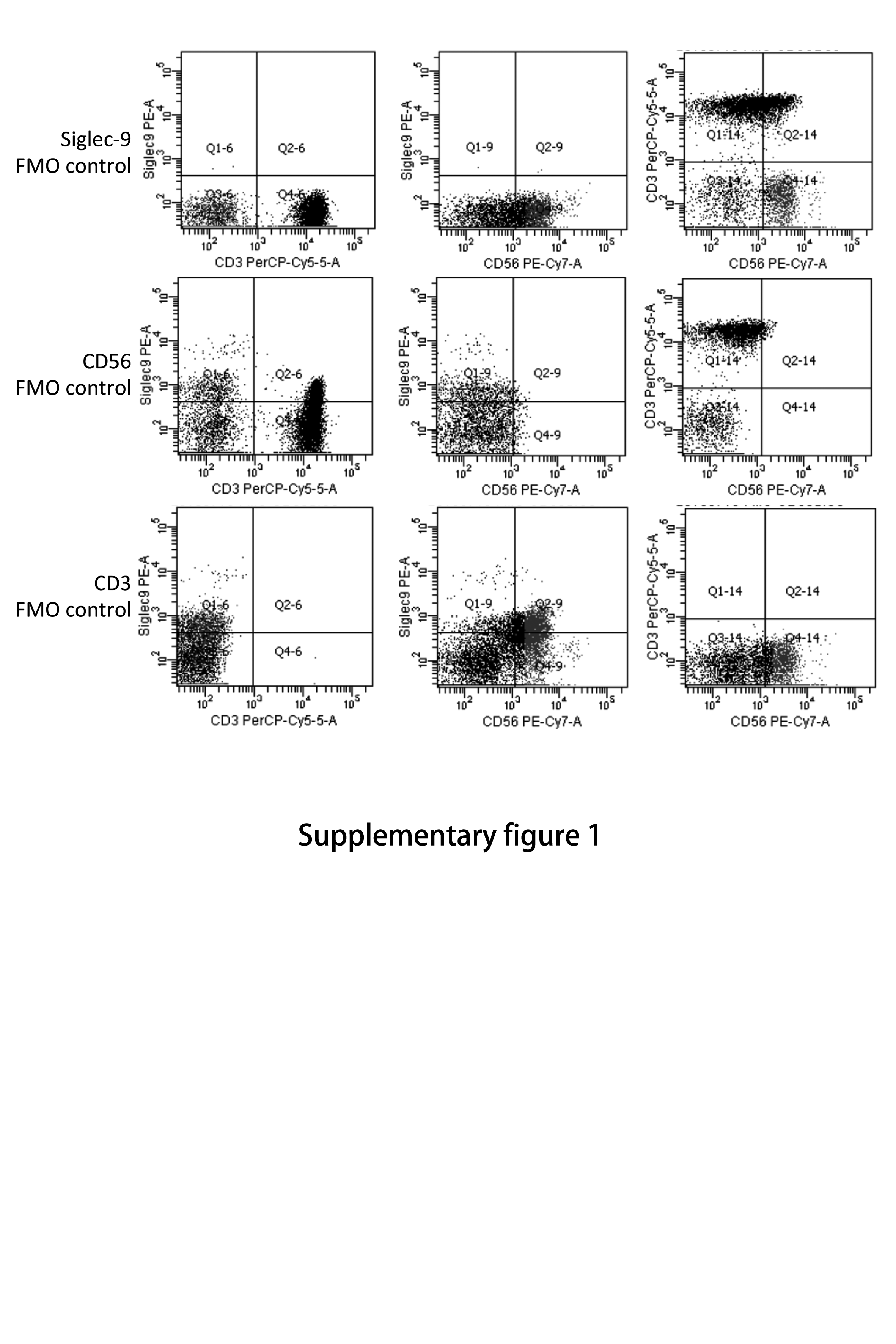

Supplement: Supplementary file 2 [file Image_1.TIF]

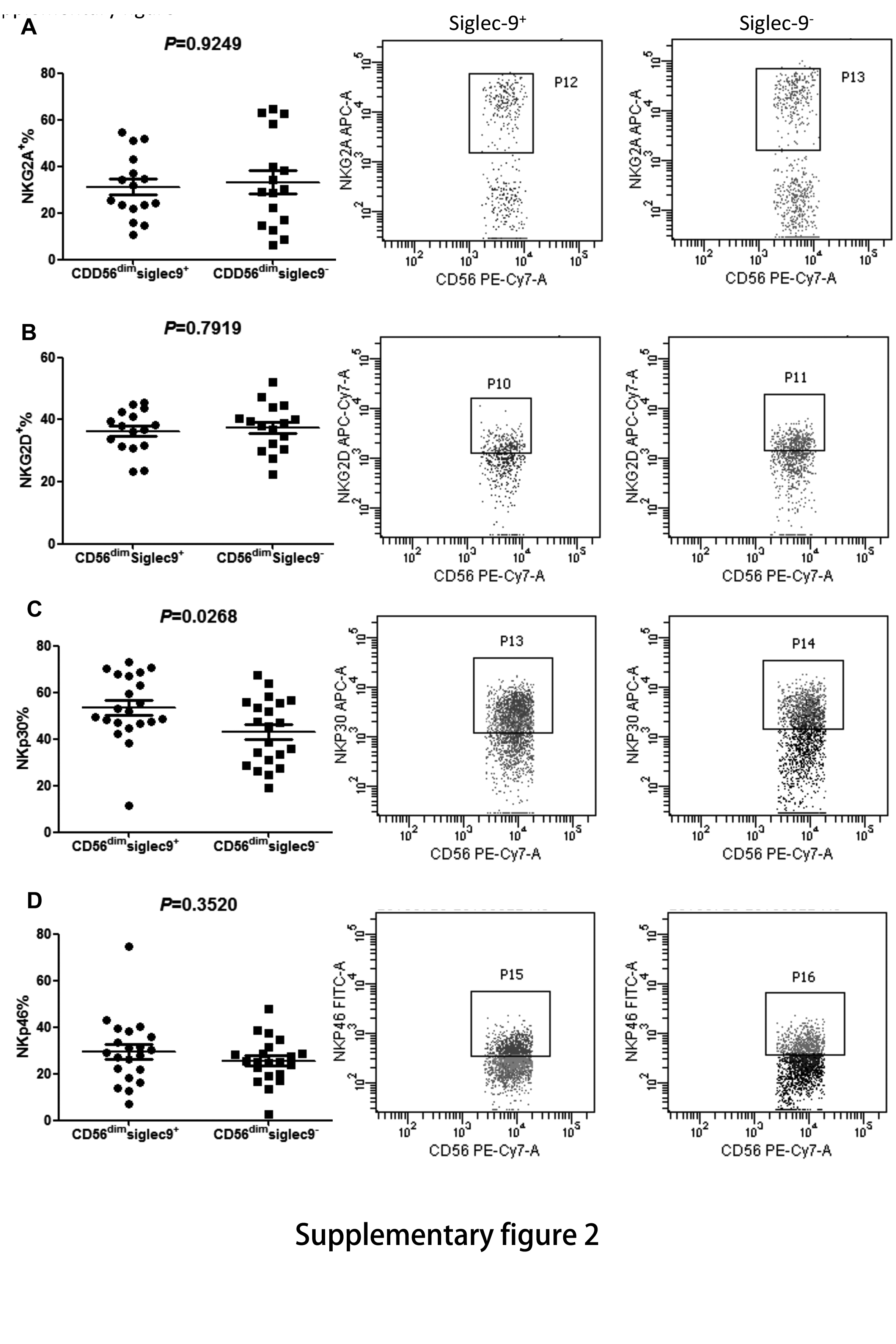

Supplement: Supplementary file 3 [file Image_2.TIF]

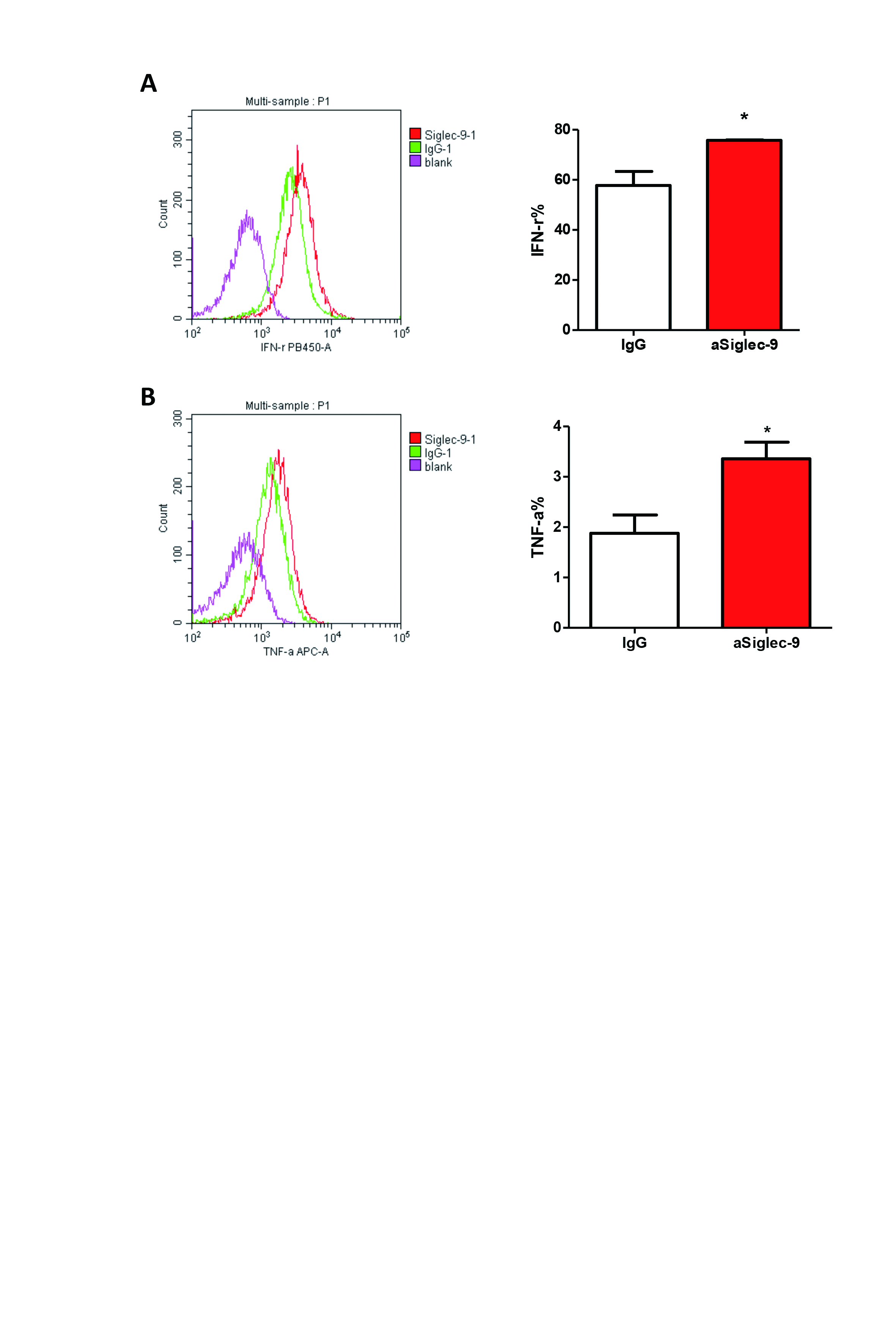

Supplement: Supplementary file 4 [file Image_3.TIF]

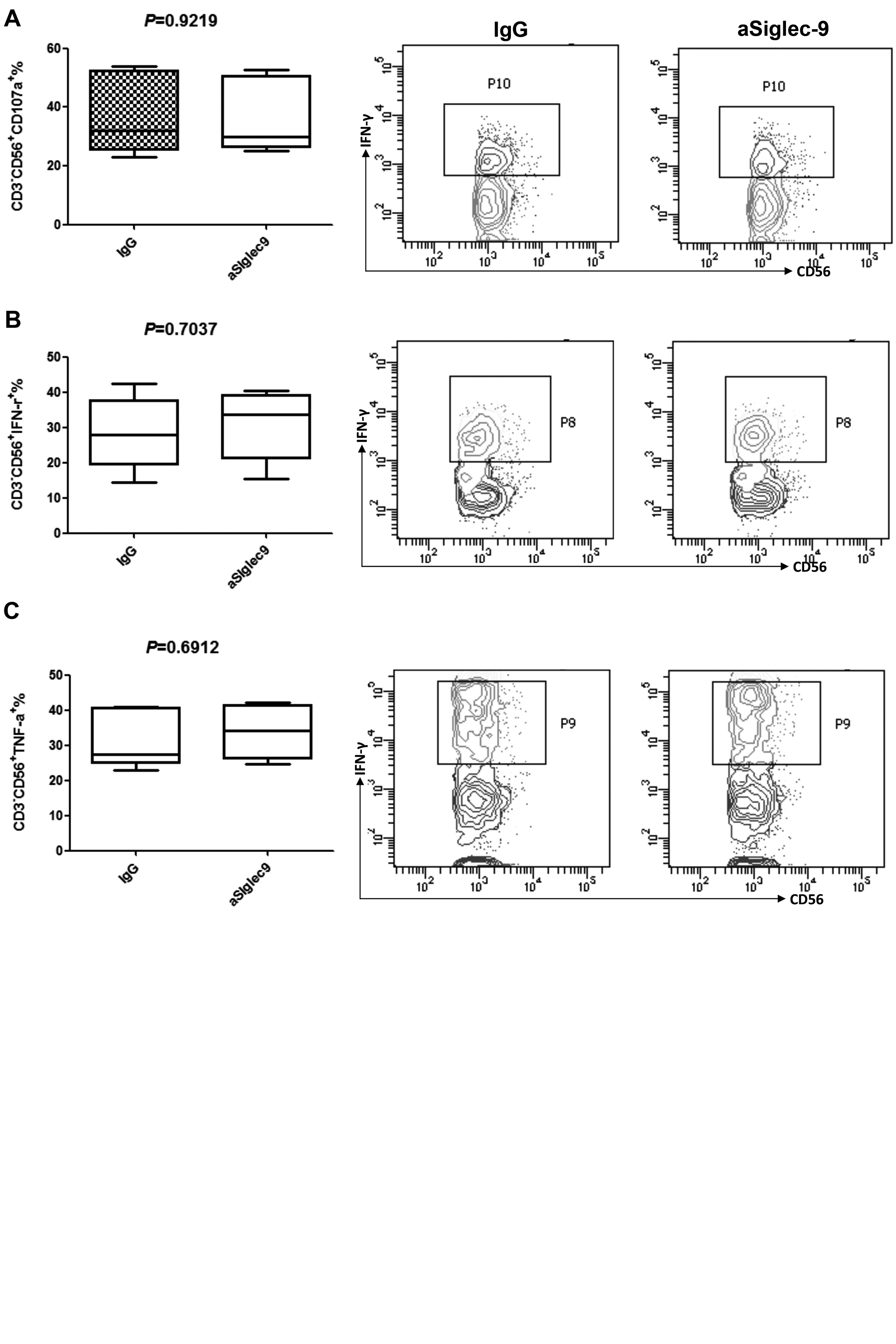

Supplement: Supplementary file 5 [file Image_4.TIF]

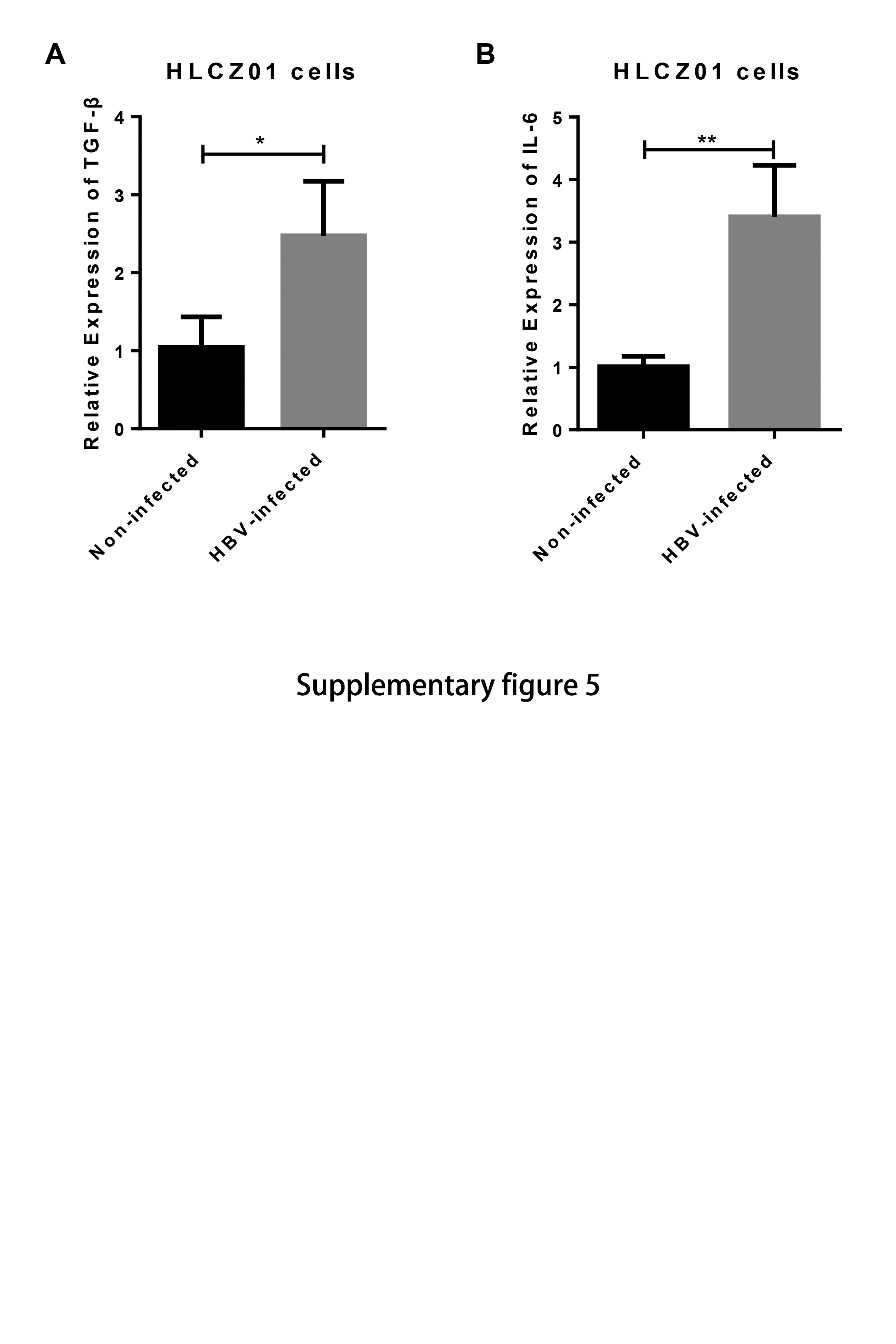

Supplement: Supplementary file 6 [file Image_5.TIF]

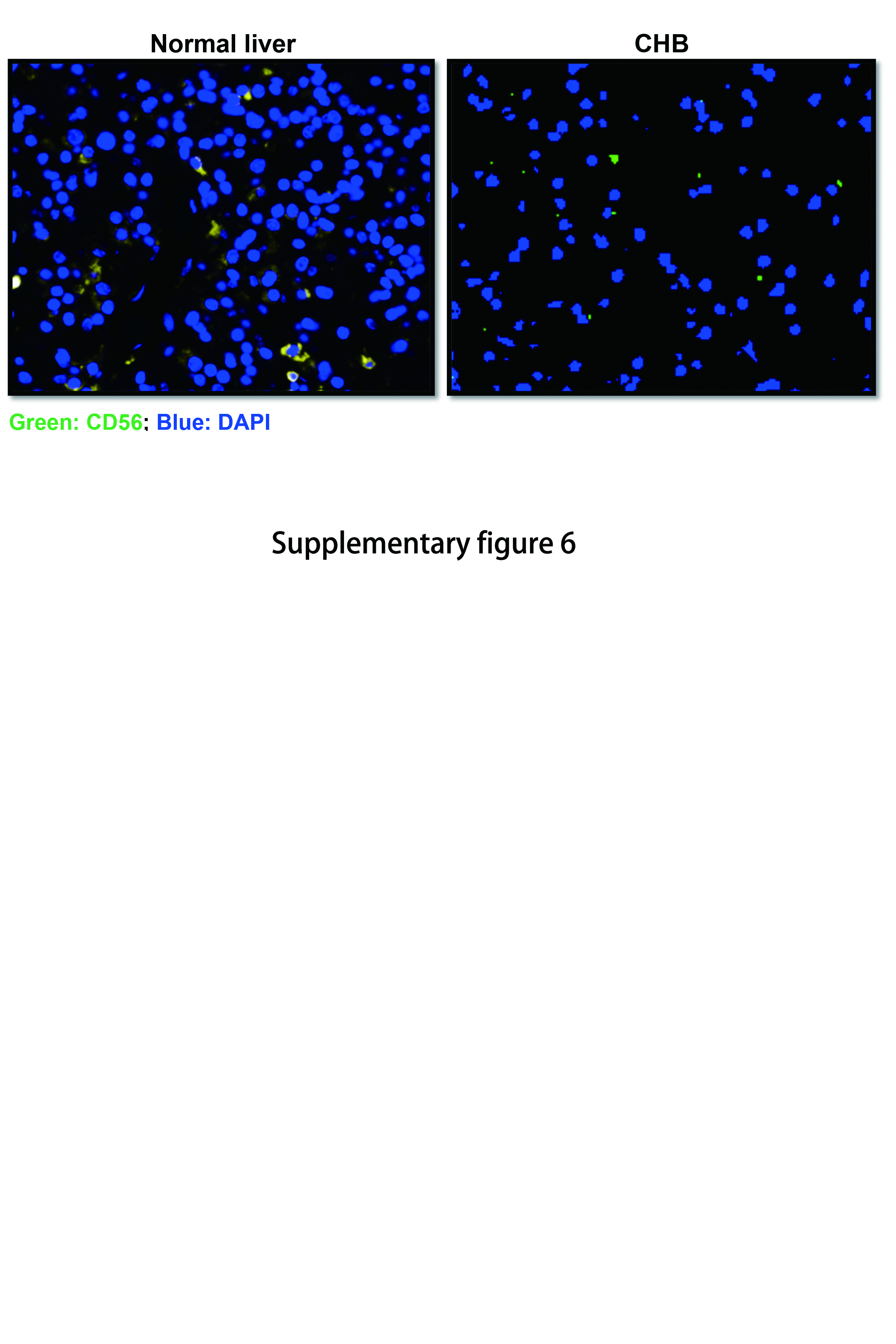

Supplement: Supplementary file 7 [file Image_6.TIF]
